# Consumer perspectives on grab bars: A Canadian national survey of grab bar acceptability in homes

**DOI:** 10.3389/fpubh.2022.915100

**Published:** 2022-10-17

**Authors:** Iris C. Levine, Sin-Tung Lau, Emily C. King, Alison C. Novak

**Affiliations:** ^1^KITE-Toronto Rehabilitation Institute, University Health Network, Toronto, ON, Canada; ^2^Dalla Lana School of Public Health, University of Toronto, Toronto, ON, Canada; ^3^VHA Home HealthCare, Toronto, ON, Canada; ^4^Rehabilitation Sciences Institute, University of Toronto, Toronto, ON, Canada; ^5^Faculty of Kinesiology and Physical Education, University of Toronto, Toronto, ON, Canada

**Keywords:** building codes (standards), safety, accessibility, aging-in-place, Universal Design (UD)

## Abstract

Given the prevalence and severity of bathroom falls and injuries across age groups, there is growing interest in policy-level approaches to bathroom fall prevention. Grab bars reduce fall risk during bathing transfers and improve bathing accessibility for adults of all ages and abilities. However, they are frequently absent from bathing environments, even in the homes of individuals who have a specific need for a grab bar. While mandatory bathroom grab bar installation has been suggested, it is unclear whether this would be supported by Canadians. The purpose of this study was to characterize Canadian public perceptions on the installation and use of grab bars in home bathrooms. We surveyed 443 Canadians about whether they currently had a grab bar and their perspectives on grab bar policy. 65.4% of respondents did not have a grab bar. However, 88.5% of respondents would allow a grab bar to be installed in their bathroom at no cost to them, only 11.5% of respondents would object to grab bar installation becoming mandatory in new builds, and 85.6% of respondents would use a grab bar if it were installed in their bathroom. Responses were affected by age (in four groups: 18–39, 40–59, 60–79, and 80+ years), self-reported impairment, and home ownership status. Older adults, respondents who reported having impairments, and home owners were more likely to respond favorably toward grab bars. Based on these results, the majority of Canadians would respond positively to policy mandating bathroom grab bars in new homes.

## Introduction

The design of the built environment plays a critical role in public health and safety (1). Inversely, public health advocacy plays an important role in development of evidence-based, health-focused building codes and standards (2). As an example of this, while bathrooms are essential in daily life, environmental features of the bathroom pose the greatest fall risk when compared to other rooms in the home (3). In the United States, bathtubs and showers ranked as the 8^th^ leading product involved in non-fatal injury, associated with an annual cost of nearly $20 billion (4). Grab bars in the bathtub and shower reduce injury and fatality risk through multiple avenues: reduction of fall hazard during bathing transfers, even in younger adults (5, 6); improved bathing independence and accessibility (7, 8); and reduction of occupational injury risk for caregivers in addition to the direct user (9). Beyond injury prevention, aging-in-place and independent living require the ability to manage personal hygiene activities safely. The earliest-onset challenge to this ability is often difficulty bathing (10), which is a primary reason for requiring costly home care services (11).

While falls in the bathroom are often considered to be an issue for older adults, younger adults and children also experience a high risk of injury (8, 12, 13). Echoing this, grab bars are often considered to be a component of the bathroom environment that is appropriate only for older adults or people with disabilities, which is reflected in three common reasons for not having a grab bar: perceived stigma associated with assistive devices (14, 15), perception that a grab bar is unnecessary (16), and a desire for normalcy and avoidance of permanent changes (17). As a result, grab bars are frequently absent from bathing environments, even in the homes of individuals who have been identified as having a specific need for a grab bar (18, 19). Factors such as younger age, and not having a distinct event such as hospitalization or knee or hip injury are risk factors for unmet need for bathing assistive devices (19), indicating that mismatched perceptions of utility, ability, ageism and ableism contribute to the absence of necessary grab bars. As a public health intervention to address the prevalence of preventable bathing-related falls, the Universal Design approach of including of grab bars in all bathing environments may circumvent these concerns by shifting perceptions of grab bars to that of a typical bathroom element, rather than a stigmatized assistive device. However, it is unknown what proportion of Canadians currently have a grab bar in their bathroom, would be receptive to its installation, or whether they would use a grab bar if it were installed in their bathroom.

A further potential benefit of the public health approach to grab bar installation is its potential to pre-empt common installation-related barriers experienced by those with an identified a need or desire for grab bars. Cost of installation (14) and environmental barriers to installation such as inappropriate wall materials (15) can be major obstacles to having a grab bar in a home bathroom. Members of low-income (20), marginalized (21), and older adult communities (22) have been identified as a groups that would benefit from access to grab bars on a policy level. While grab bars are addressed in accessibility standards such as CSA B651 (23) and ICC A117.1 (24), there are no existing universal requirements for builders or building owners to include grab bars, or appropriately-placed wall materials that could support grab bars, in bathrooms. Retrofitting a bathroom to support grab bar installation can be challenging due to decisions made in the initial design and construction, such as the absence of appropriate backing materials to support grab bar installation, or use of all-in-one style shower surrounds that cannot safely be modified to permit grab bar installation. Policy toward proactive inclusion of grab bars in initial phases of bathroom design would resolve these barriers by eliminating renovation costs, increasing the supply of grab-bar-ready bathtub and shower designs and molds, and including grab bars as a standard bathroom fixture across all qualities and costs of construction. While building code changes do not necessitate immediate remediation, as older buildings and units reach their end of lifespan, renovation or replacement to meet current building codes would increase availability of safer bathing environments over time. Additionally, normalization of grab bars in building policy may encourage regions to include grab bars in property standards associated with property rental licensing. Finally, normalization of grab bars in bathrooms, and greater availability of grab bars on the market may make property owners more amenable to grab bar installation, even for situations where a grab bar is not required.

While the question of mandatory installation of grab bars has met the attention of National Building Code of Canada (25), it is unclear whether the mandatory installation of grab bars would be supported by Canadians. Similar to the inclusion of patient partners in development of health policy, incorporation of public perspective is included in code development and review by mandate (26, 27). The public review phase influences whether changes move forward, are deferred or withdrawn. Code development committees are therefore influenced in their deliberations by their interpretation of public readiness to accept given code changes. However, there are barriers to participating in the formal code development and review process, such as a lack of transparency, and often limited involvement from those who are not keenly tracking the progress of the proposed code change (28). Therefore, it is helpful to understand public perspectives from a research lens in addition to formal review processes. Additionally, formal building code reviews only evaluate perspectives on mandatory changes to a home, not voluntary modification. Understanding perspectives on distinctions between mandatory and voluntary home modification is valuable for developing health-focused policies beyond the building code.

The objective of this survey was to characterize Canadian public perceptions on the installation and use of grab bars in the home. Our primary aim was to determine what proportion of a sample of Canadian adults had a grab bar installed in their bathroom, and what demographic characteristics differed between those who did and did not have a grab bar installed. We also aimed to determine whether there would be support for mandating grab bar installation in residential construction, and to evaluate what demographic factors played a role in these opinions.

## Materials and methods

The procedure for this study was approved by the Research Ethics Board of the University Health Network, Toronto, ON, Canada. Methods were carried out according to relevant guidelines and standards. All participants provided electronic informed consent. Between September 2019 and September 2020 (52 weeks), Canadian adults were recruited nationally *via* newspaper and digital advertisements which contained a permanent weblink to the survey hosted at SurveyMonkey^®^ (https://www.surveymonkey.com/, SurveyMonkey, Inc., San Mateo, USA). Study advertisements were delivered across Canada in printed newspapers and as digital ads on news and other content websites. We did not predetermine who would be served the printed or digital ads; the printed advertisements appeared in several national and local newspapers across Canada. The digital advertisements were delivered across a period of 14 days and appeared on websites in a random fashion over the time period. No specific group or individual was targeted for recruitment and survey respondents were recruited *via* convenience sampling. To participate in the survey, respondents must have self-reported to be a Canadian resident, over the age of 18 years old. There were no health-related inclusion or exclusion criteria for this study. Respondents were given the opportunity to contact the research team *via* phone or email to access the survey by phone or receive a paper copy of the survey. The survey was provided in English language only.

### Survey content

Respondents completed a sixteen-question survey (see Appendix), with all questions posed to each participant in the same order. Survey questions collected information related to participant demographics, disability status, living environment, and a set of grab-bar-specific questions. While respondents were not required to answer all questions, their responses were excluded if they did not respond to at least one of the four grab-bar-specific questions. Specifically, respondents reported their age (numerical), gender (woman; man; other), and primary job sector (according to North American Industry Classification System categories). Regarding disability status, respondents reported impairment with mobility, vision/seeing, hearing or cognition. Regarding living environment, respondents reported home ownership status (rent; own; neither), home type (detached, semi-detached or row house; apartment or condominium; mobile home or trailer). Specific to grab bar use and acceptability, participants were asked:

(GB1) Do you have a grab bar in your bathroom?

(GB2) Would you permit a grab bar to be installed if it was provided at no cost to you?

(GB3) Would you OBJECT to grab bar installation becoming mandatory in newly built or newly renovated homes?

(GB4) Would you use a grab bar if it was in your bathroom?

For the grab-bar-specific questions, respondents selected from “yes”, “no”, “prefer not to answer” or skipped the question.

### Data analysis

Data were analyzed using SPSS (Version 22, IBM Corp., Armonk, USA). For all questions, “prefer not to answer”, “other”, skipped questions, or demographic factors with <10 responses (e.g., some job sectors represented by few respondents) were all categorized as “no response” to avoid biasing results by low response numbers in each of these categories. Age was treated as both a numerical response and binned into 18–39, 40–59, 60–79, and 80+ categorical age groups. Regarding the first objective, we evaluated the frequency of each response to GB1. We used X^2^ to evaluate whether any demographic factors differed between respondents who did or did not have a grab bar in their bathroom. In support of the second objective, we evaluated the frequency of response to GB2, GB3, and GB4. We used X^2^ to evaluate whether any demographic factors differed between responses for GB2, GB3, and GB4. In consideration of potential age confounds, we also projected anticipated responses by correcting the proportional representation of each age group to the proportion reported by Statistics Canada (29). For the second objective, we anticipated that respondents who currently have a grab bar in their bathroom might respond more positively to installation and use of grab bars than those who did not have a grab bar. Accordingly, we treated the response to GB1 as an additional potential demographic factor. In all cases where X^2^ results were significant, we calculated risk ratios (RR) and confidence intervals to estimate the magnitude of the effect. Finally, we evaluated the characteristics of participants who responded “Yes” to GB2 and GB3 (i.e., those who would accept voluntary installation of a grab bar, but not mandatory installation of a grab bar) *via* a *t*-test (for age) and RR (for other characteristics. All tests employed a univariate approach, and statistical significance was considered at *p* < 0.05.

## Results

Of the 519 respondents who initiated the survey, 443 (85.4%) completed the survey and were included in this analysis; 27 (5.2%) respondents provided demographic, disability status and/or living environment information, but did not answer the grab-bar-specific questions, and 49 (9.4%) did not self-report as meeting inclusion criteria or did not consent to participate. Only participants who completed the study were included in the analysis.

### Respondent demographics

The mean (SD) age of respondents was 56.8 (16.1) years. 63.8% of respondents identified themselves as women, however, responses to the four grab bar questions did not differ between men, women or participants who did not select “man” or “woman”. Respondent demographics are presented in Table 1, along with comparative Canadian data. Overall frequency of responses to each question, divided by demographic, disability status and living environment are presented in Table 2.

**Table 1:** Participant demographics, disability status and living environment.

	**Frequency**	**Percent**	**Canada (%)**
**Demographic**			
**Age**			
18–39	76	17.1	36.8^a^
40–59	129	29.1	32.7^a^
60–79	215	48.5	25.1^a^
80+	24	5.4	5.4^a^
**Gender**			
Woman	284	63.8	50.6^a^
Man	153	34.4	49.4^a^
Other or no response	6	1.25	–^b^
**Job sector**			
Agriculture, forestry, fishing and hunting, natural resource extraction	11	2.5	1.6^c^
Utilities	10	2.3	0.8^c^
Construction	12	2.7	7.6^c^
Manufacturing	17	3.8	9.2^c^
Wholesale and retail trade	32	7.2	14.9^c^
Transportation and warehousing	14	3.1	5.3^c^
Information and cultural industries, arts, and entertainment and recreation	40	9.0	3.8^c^
Finance and insurance, real estate and rental and leasing	36	8.1	6.9^c^
Professional, scientific and technical services	70	15.7	8.5^c^
Management of companies and enterprises	21	4.7	3.9^c^^d^
Education services	64	14.4	7.4^c^
Health care and social assistance	77	17.3	13.5^c^
Public administration	25	5.6	5.5^c^
Other job sectors with <10 responses, no response, or no job sector specified	14	3.2	–^b^
**Disability status**			
Any impairment, yes	295	66.6	22.3^e^
Mobility impairment, yes	142	31.9	9.6^e^
Vision/Seeing impairment, yes	175	39.3	5.4^e^
Hearing impairment, yes	50	11.2	4.8^e^
Cognitive impairment, yes	16	3.6	–^b^
**Living environment**			
**Home ownership status**			
Rent	84	18.9	–^b^
Own	337	75.7	69.0^f^
Other, neither, or no response	22	5.0	–^b^
**Dwelling type**			
House (including detached, semi-detached or row house)	315	70.8	66.2^g^
Apartment or condominium	120	26.8	32.4^g^
Other, mobile home or trailer, or no response	8	1.8	–^b^

a (29).

b Data unavailable.

c (35).

d Category renamed to “Business, building and other support services.

e (32).

f (36).

g (37).

**Table 2:** Frequency of question responses (%), by demographic factors, disability status, and living environment.

	**Currently have in home**			**Would allow at no cost**			**Would object if mandatory**			**Would use if installed**		
**Group**	**Yes**	**No**	**Other^a^**	**Yes**	**No**	**Other^a^**	**Yes**	**No**	**Other^a^**	**Yes**	**No**	**Other^a^**
**Gender**												
Men	33.99	66.01	0.00	86.27	10.46	3.27	16.34	81.70	1.96	83.66	16.34	0.00
Women	34.04	65.61	0.35	89.82	7.02	3.16	8.42	90.18	1.40	87.37	12.28	0.35
Other^a^	33.33	66.67	0.00	100.00	0.00	0.00	33.33	66.67	0.00	83.33	16.67	0.00
**Age**												
18–39	23.68	76.32	0.00	80.26	18.42	1.32	23.68	73.68	2.63	72.37	27.63	0.00
40–59	22.48	77.52	0.00	82.17	10.85	6.98	12.40	86.82	0.78	79.07	20.16	0.78
60–79	39.53	60.00	0.47	94.88	3.26	1.86	6.98	91.16	1.86	93.49	6.51	0.00
80+	79.17	20.83	0.00	95.83	4.17	0.00	8.33	91.67	0.00	100.00	0.00	0.00
**Impairment**												
Any	39.32	60.68	0.00	91.19	6.10	2.71	11.53	86.44	2.03	92.20	7.80	0.00
Mobility	48.98	51.02	0.00	94.56	4.08	1.36	11.56	85.03	3.40	97.28	2.72	0.00
Vision	32.39	67.61	0.00	89.77	6.82	3.41	10.80	87.50	1.70	89.20	10.80	0.00
Hearing	46.00	54.00	0.00	94.00	2.00	4.00	14.00	86.00	0.00	96.00	4.00	0.00
Cognitive	41.18	58.82	0.00	100.00	0.00	0.00	23.53	76.47	0.00	100.00	0.00	0.00
**Environment**												
Rent	47.67	51.16	1.16	89.53	6.98	3.49	15.12	82.56	2.33	94.19	5.81	0.00
Own	31.36	68.64	0.00	88.76	8.28	2.96	10.65	88.17	1.18	83.73	15.98	0.30
Other^a^	20.00	80.00	0.00	85.00	10.00	5.00	10.00	85.00	5.00	90.00	10.00	0.00
House (including detached, semi-detached or row house)	47.54	52.46	0.00	94.26	4.10	1.64	13.11	84.43	2.46	95.08	4.92	0.00
Apartment or condominium	28.89	70.79	0.32	86.35	9.84	3.81	11.11	87.62	1.27	82.86	16.83	0.32
Other^a^	28.57	71.43	0.00	100.00	0.00	0.00	0.00	100.00	0.00	71.43	28.57	0.00

a Includes no response, “other”, “prefer not to say”, responses, and responses with frequency <5 responses.

**Figure 1:**
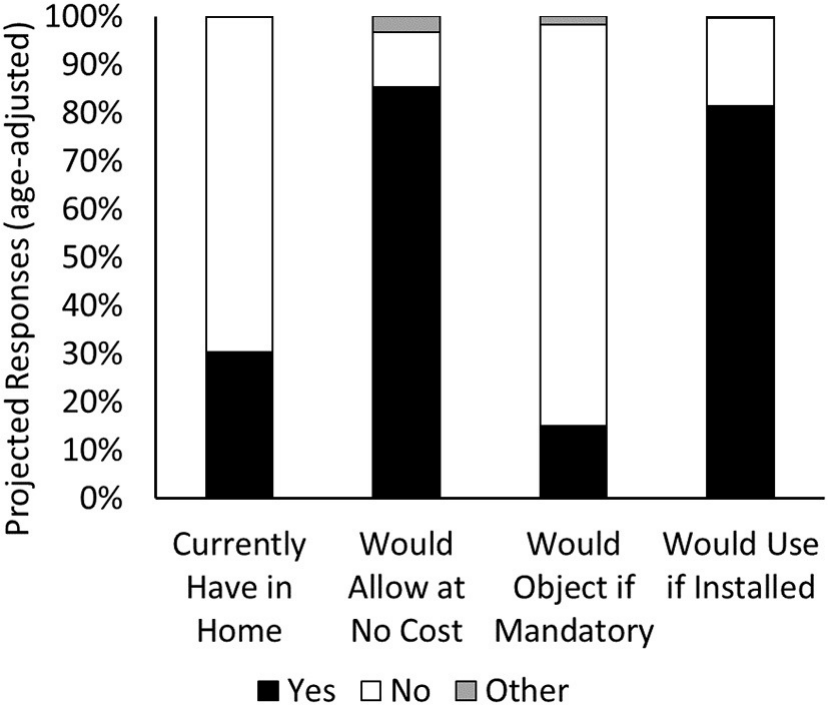
Overall responses to questions GB1-4, age-adjusted. While most respondents did not have a grab bar in their home bathroom, most responded that they would allow a grab bar to be installed in their home, would not object to mandatory grab bar installation, and would use a grab bar if it were installed in their home.

Regarding the first objective, 65.4% of all respondents did not currently have a grab bar in their bathroom, while 33.7% of respondents did. When adjusting for age [relative to the proportion of the Canadian population in each of our age groups (29)], only 30.3% of respondents had a grab bar in their home (Figure 1). Respondents in the 80+ year groups were most likely to already have a grab bar installed in their bathroom, followed by the 60–79 group (Table 3). Respondents were more likely to have a grab bar if they reported having any impairment or mobility impairment. Homeowners were more likely to have a grab bar installed than renters, while apartment or condo dwellers were more likely to have a grab bar than respondents who lived in a detached, semi-detached or row house. While responses differed between job sectors, no specific job sector was more or less likely to have a grab bar when compared to all other sectors combined.

**Table 3:** Probability of currently having a grab bar (GB1): Comparison by respondent demographic characteristics, disability status and living environment.

**Factor**	**X^2^**	***p***	**Groupwise comparisons**		**RR**	**95% CI**
Gender	2.9	0.816				
Age group	37.3	<0.001^*^	80+ vs.	18–39	3.34	2.13–5.26
				40–59	3.52	2.41–5.15
				60–79	1.99	1.53–2.59
			6–79 vs.	18–39	1.68	1.08–2.59
				40–59	1.77	1.23–2.53
Job sector	97.5	<0.001^*^				
Any impairment	12.7	0.002^*^	Any impairment vs.	No impairment	1.66	1.20–2.30
Mobility impairment	22.6	<0.001^*^	Mobility impairment vs.	No mobility impairment	1.83	1.42–2.35
Vision impairment	1.0	0.596				
Hearing impairment	3.7	0.158				
Cognitive impairment	0.4	0.804				
Home ownership status	15.6	0.006^*^	Renters vs.	Home owners	0.66	0.50–0.87
Dwelling type	14.1	0.029^*^	Apartment or condominium vs.	Detached, semi-detached or row house	1.64	1.27–2.12

Only significant risk ratios between comparative groups have been included in this table RR = risk ratio 95% CI = 95% Confidence Interval.

* Indicates significant X^2^ results at *p* < 0.05.

Regarding the second objective, 88.5% (age-adjusted, 85.4%) of respondents would allow a grab bar to be installed in their bathroom at no cost to them and only 11.5% (age-adjusted, 15%) of respondents reported that they would object to grab bar installation becoming mandatory in new builds. Only Age Group significantly influenced either of these responses, with respondents 60–79 years being more likely to allow a grab bar to be installed and less likely to object to a mandatory grab bar than respondents 18–49 years (Tables 4, 5). Only while there was a difference in response to GB2 between respondents who self-reported a mobility impairment and those who did not, this did not result in a difference in the probability of objecting to mandatory grab bar installation (GB3). Finally, 85.6% (age-adjusted, 81.4%) of respondents would use a grab bar if it were installed (GB4). Respondents older than 80 years were most likely to use a grab bar, followed by those 60–79 years, followed by both 18–39 and 40–59 year age groups (Table 6). Those who reported having mobility impairment were more likely to state that they would use a grab bar than those who did not report having any mobility impairment; however, respondents who reported having any impairment (including mobility, vision, hearing and cognitive impairments) were less likely to state that they would use a grab bar than those who did not report having any impairment. Respondents who currently had a grab bar installed in their bathroom were more likely to state that they would use a grab bar than those who did not have a grab bar installed.

**Table 4:** Probability of allowing a grab bar at no cost (GB2): Comparison by respondent demographic characteristics, disability status and living environment.

**Factor**	**X^2^**	***p***	**Groupwise comparisons**		**RR**	**95% CI**
Gender	3.0	0.965				
Age group	31.6	<0.001^*^	60–79 vs.	18–39	1.09	1.01–1.17
Job sector	47.2	0.932				
Any impairment	6.4	0.094				
Mobility impairment	7.8	0.255				
Vision impairment	2.2	0.539				
Hearing impairment	2.1	0.374				
Cognitive impairment	2.2	0.523				
Home ownership status	4.8	0.565				
Dwelling type	10.4	0.318				
Currently have grab bar	1.5	0.957				

Only significant risk ratios between comparative groups have been included in this table RR = risk ratio 95% CI = 95% Confidence Interval.

* Indicates significant X^2^ results at *p* < 0.05.

**Table 5:** Probability of objecting to mandatory grab bar installation (GB3): Comparison by respondent demographic characteristics, disability status and living environment.

**Factor**	**X^2^**	***p***	**Groupwise comparisons**		**RR**	**95% CI**
Gender	11.6	0.235				
Age group	18.6	0.029^*^	60–79 vs.	18–39	0.30	0.15–0.59
Job sector	55.9	0.725				
Any impairment	1.3	0.729				
Mobility impairment	12.9	0.045^*^				
Vision impairment	1.1	0.787				
Hearing impairment	1.2	0.752				
Cognitive impairment	2.7	0.435				
Home ownership status	4.2	0.898				
Dwelling type	4.2	0.898				
Currently have grab bar	1.5	0.957				

Only significant risk ratios between comparative groups have been included in this table RR = risk ratio 95% CI = 95% Confidence Interval.

* Indicates significant X^2^ results at *p* < 0.05.

**Table 6:** Probability of using a grab bar installed in the home (GB4): Comparison by respondent demographic characteristics, disability status and living environment.

**Factor**	**X^2^**	***p***	**Groupwise comparisons**		**RR**	**95% CI**
Gender	2.2	0.897				
Age group	32.8	<0.001	80+ vs	18–39	1.17	1.07–1.29
				40–59	1.20	1.11–1.30
				60–79	1.05	1.02–1.09
			60–79 vs.	18–39	1.11	1.01–1.23
				40–59	1.14	1.05–1.24
Job sector	21.8	0.996				
Any impairment	28.5	<0.001^*^	Any impairment vs.	No impairment	0.81	0.73–0.89
Mobility impairment	23.5	<0.001^*^	Mobility impairment vs.	No mobility impairment	1.21	1.13–1.28
Vision impairment	2.8	0.242				
Hearing impairment	4.6	0.097				
Cognitive impairment	2.9	0.238				
Home ownership status	6.7	0.159				
Dwelling type	12.4	0.053				
Currently have grab bar	20.0	<0.001^*^	Currently have a grab bar	Currently do not have grab bar	1.21	1.14–1.29

Only significant risk ratios between comparative groups have been included in this table RR = risk ratio 95% CI = 95% Confidence Interval.

* Indicates significant X^2^ results at *p* < 0.05.

Participants who responded “Yes” to GB2 and GB3 were younger [mean (SD) age = 49.6 (18.5); *t* = 2.4, *p* = 0.011], and more likely to identify as a man (RR = 1.55, CI = 1.09–2.20), having a cognitive impairment (RR = 3.58, CI = 1.23–10.39) and/or dwelling in a house (RR = 2.12, CI = 1.50–3.00) than other respondents. We did not find statistically significant differences between the 35 participants (7.84% of the sample) in this subgroup and other respondents in self-reported overall impairment, mobility impairment, vision impairment, hearing impairment or home ownership status.

## Discussion

We aimed to evaluate Canadian public perceptions on the installation and use of grab bars in the home. Despite grab bars only being installed in a third of Canadian homes, we found that respondents stated overwhelmingly that they would allow a grab bar to be installed in their bathroom and use a grab bar if it were installed, and did not object to grab bar installation becoming mandatory in newly built or newly renovated homes. Opinions were most strongly affected by age group and whether the individual had a mobility impairment; whether or not respondents already had a grab bar installed was also influenced by home ownership status and dwelling type. This is the first study to characterize the current frequency of grab bars in Canadian homes, and the segments of the population who are at greatest risk of not having a grab bar in their bathroom. Additionally, our study provides insight into perspectives on voluntary and mandatory inclusion of grab bars, which has importance for developing policy and programs focused on the safety of the home environment.

Age affected responses to all four questions, with older adults responding more frequently than younger adults that they currently had a grab bar installed, would allow a grab bar to be installed, would not object to mandatory grab bar installation, and would use a grab bar. This was expected, as older adults are the typical age range for whom a grab bar is commonly recommended. However, grab bars are likely to also be effective for younger adults (5, 6, 30), not only in fall prevention, but also potentially in caretaker roles (9), or during periods of temporary mobility impairment such as illness or fatigue. Despite this potential age effect, 72.3% of respondents in the youngest group stated that they would use a grab bar if it were installed in their home, and only 23.7% stated they would object to mandatory grab bar installation. The respondent sample was biased toward the 60–79 year age group relative to the general population of Canada (29). However, even when adjusted for age, support for grab bars remained strong. Based on the analysis of age groups and high acceptance rate noted for the younger adults included in this study, this bias was not likely to affect the overall acceptance of grab bars or objection to mandatory grab bars. The value of grab bars seems to be apparent to the majority of respondents across all age groups.

However, from a policy perspective, there was a small group of who would both allow a grab bar to be installed, but would object to mandatory requirement of grab bars. While we do not wish to over-interpret the characteristics of this subgroup, it would be valuable to understand the perspectives of the subgroup and the how these perspectives should be considered in development of mandatory programs, such as building policy and voluntary programs aimed at improving home safety. More robust study of this subgroup, along with others, would help to develop multifaceted approaches to improving safety-prioritized home modification. However, this group represents only a small portion of the overall study population, limiting the effect on the overall study results. Further, percentage of positive responses (age-adjusted) to the question of voluntary installation, and negative responses to the question of mandatory installation were separated by only 2.1% points, suggesting that both mandatory and voluntary strategies would have similar levels of public support.

Similarly, self-reported impairment, particularly mobility impairment, affected the likelihood of respondents stating that they already had a grab bar installed, or would use a grab bar if they had one. However, the presence or absence of a self-reported impairment did not affect whether they would allow a grab bar to be installed, or whether they would object to mandatory grab bar installation. It is promising from a policy and Universal Design standpoint that grab bars are viewed positively by people with and without disabilities, rather than being favored only by those with the greatest immediate need. It should be noted that our definitions of impairment were broad, which may have affected the responses to each question. For example, we included balance, using stairs and using an assistive mobility device within “mobility impairment”, however, the effectiveness of grab bars for providing mobility-specific support may vary depending on each person's specific mobility impairment(s). Similarly, we included corrective lens wearers within “vision impairment” because we anticipated that these respondents would not wear their corrective lenses while bathing. The United States FDA advises against wearing contact lenses during bathing (31), and glasses wearers may find that water droplets from a shower may negate the effectiveness of their glasses. However, the visual acuity of corrective lens wearers is extremely variable, which may have washed out more strongly positive opinions by respondents with very low or no vision, for whom the proprioceptive benefits of a grab bar may be greater than for respondents with more moderate vision impairment. Differences in definitions of disability likely also explains differences in prevalence of each self-reported disability, and national prevalence reported by Statistics Canada (32), which relies on more stringent definitions of professionally-diagnosed impairments. For example, the prevalence of vision impairment reported by Statistics Canada is 5.4% (Table 1), while the prevalence in the current study was 39.3%. However, 86.1% of Canadians report wearing corrective lenses (33), representing a portion of the population which may experience greater visual impairment during bathing than other tasks in their daily life.

We found that respondents who currently had a grab bar installed in their home were more likely to state that they would use a grab bar; however, despite positive attitudes toward grab bars, only a third of respondents currently have a grab bar in their home, indicating a passive mismatch between openness to and perceived utility of a grab bar, but not having a grab bar installed. Renters were less likely to have a grab bar installed than homeowners. Renting has previously been cited as a risk factor for not having a grab bar, as many renters are discouraged or prohibited from making permanent changes to their living space, and landlords may not value the benefits of grab bars over the cost of installation (15, 22). Our findings thus highlight the potential value of a policy mandating grab bars installation, which would allow renters similar access to grab bars as homeowners. Apartment or condominium dwellers were more likely to have a grab bar installed than house dwellers. Although we cannot be certain why based on available data, it is possible that these individuals were benefitting from apartments and condominium units designed specifically for older adults. We found that respondents who currently had a grab bar installed in their home were more likely to state that they would use a grab bar. It is likely that people who perceive a need or use for a grab bar are more likely to install one. However, it is also possible that once a grab bar is installed (e.g., if one moves into a home where a grab bar has already been installed), the perceived use of the grab bar may increase. Accounting for this mismatch, it is our recommendation that architects and designers include, at minimum, appropriate wall backing for grab bars in all residential designs. Further, we anticipate that inclusion of grab bars as standard procedure in bathroom design may be welcomed by home building and renovating clients. While we specifically asked respondents whether they would allow a grab bar to be installed at no cost, we recognize that this is unrealistic. However, overall positive perceptions suggest that clients may not object to the relatively low cost of installing a grab bar compared to the overall cost of building a new dwelling or renovating a bathroom.

This study has several limitations. First, the survey was conducted entirely online. Responses may have been biased against potential respondents who blocked advertisements, were not served an online advertisement due to lack of time spent surfing the internet, or lived in communities where internet access is limited. Recent Statistics Canada data indicate that 94% of Canadians have household internet access (34) and most use the internet daily. While the study was conducted entirely online, we do not expect that a lack of internet access would have affected the results since the majority of Canadians have access to, and use, the internet on a regular basis. Second, given that half of the time allotted for the survey occurred during the COVID-19 pandemic, responses may have been biased away from populations for whom time available for internet-based leisure was restricted (e.g., workers in essential workforces) or for whom assistance with internet-based leisure was limited (e.g., residents of care facilities who would normally have undertaken the survey with the technical assistance of a family caregiver). While we did find that job sector affected likelihood of currently having a grab bar installed, we did not recruit enough respondents from each job sector to accurately characterize the differing perceptions of these subpopulations. In-depth analysis on the influence of job sector or level of education may provide insight into how to encourage safety-prioritized home renovation. Third, we did not conduct a multivariate analysis. While our results highlight several important factors which influence grab bar preferences, it would be valuable in future studies investigating safety-prioritized home modification to more thoroughly evaluate confounding factors, and the relative weighting of each factor. Further, causal relationships, and the relationships of the factors we evaluated, without confounds, cannot be interpreted from the analysis we have conducted. Finally, respondents to the survey may have been biased toward people who have an interest in grab bars, either positive or negative. However, we used neutral advertising statements (“Research Study: Adults (18 years old and older) wanted for online survey on bathroom grab bars”, and “We are doing a research study on bathroom grab bars. We want to learn about your views on installing and using grab bars in your bathroom.”) to limit the effect of the study advertisement on survey responses.

The majority of respondents to this study would accept a grab bar in their home and would not object to grab bars becoming mandatory in new homes or newly renovated homes. Younger individuals were less likely to accept a grab bar in their home or use a grab bar compared to older individuals; however, despite this age-related difference, the overall the acceptance rate was high across age groups, which is promising for policy development. A subgroup of younger, man-identifying respondents who reported having cognitive impairment serve as a potential group for exploration into those who perceive value from safety modification but object to policy requiring such modification. The findings from this survey provide insight into perceptions of grab bar use and acceptability by the general public in Canada, hopefully paving the way for the implementation of this public health approach to preventing bathing-related falls.

## Data availability statement

The raw data supporting the conclusions of this article will be made available by the authors, without undue reservation.

## Ethics statement

The studies involving human participants were reviewed and approved by the Research Ethics Board at the University Health Network. The patients/participants provided their written informed consent to participate in this study.

## Author contributions

AN and EK acquired funding for the project. IL, S-TL, EK, and AN developed the research question and survey material collaboratively and edited and approved of the final manuscript. IL and S-TL administered the survey. IL lead data analysis, and all analytical methods and interpretations were agreed upon by all authors. IL wrote the manuscript. All authors consent to submission of the manuscript.

## Funding

This work was supported by a Canadian Institutes of Health Research Project Grant (#159579).

## Conflict of interest

The authors declare that the research was conducted in the absence of any commercial or financial relationships that could be construed as a potential conflict of interest.

## Publisher's note

All claims expressed in this article are solely those of the authors and do not necessarily represent those of their affiliated organizations, or those of the publisher, the editors and the reviewers. Any product that may be evaluated in this article, or claim that may be made by its manufacturer, is not guaranteed or endorsed by the publisher.
