# Supplementary material for: Consumer perspectives on grab bars: A Canadian national survey of grab bar acceptability in homes
Source: Front Public Health. 2022 Oct 17;10:915100. doi: 10.3389/fpubh.2022.915100 (PMC9618860; doi:10.3389/fpubh.2022.915100)
Supplement: Supplementary file 1 [file Table_1.DOCX]

**Bathroom Grab Bar Acceptability Survey**

***Introduction***

**WHO WE ARE:**

We are a team of researchers from the Toronto Rehabilitation Institute, University Health Network in Toronto, Canada.

**ABOUT THIS SURVEY:**

Slips, trips, and falls are important safety concerns for many people when getting into and out of, and using their bathtub or shower stall. Currently, it is NOT mandatory for new home construction in Canada to include bathroom grab bars. We are interested in learning about your views on installing and using grab bars in your bathroom.

Estimated Time to Complete Survey: 3 to 5 minutes

**WHO IS INVITED TO PARTICIPATE:**

We are interested in learning from everyone who is 18 years old or older and lives in Canada. If you are interested in taking part in this survey, please confirm your age and place of residence to verify your eligibility.

**DRAW FOR GIFT CARDS:**

At the end of the survey, you have the opportunity to enter a draw for a $20 gift card. A gift card will be issued to 1 person out of every 10 people who complete the survey.

**CONTACT INFORMATION:**

Iris Levine

E-mail: **********

Telephone: **********

**ACCOMMODATIONS AND ALTERNATIVE FORMATS:**

Please contact Iris if you need accommodations to take part in the survey. For example, paper versions of this survey with text in regular 12 pt. font or in large 16 pt. font can be sent to you in the mail.

1. Are you 18 * years old or older?

- Yes
- No

* 2. Do you live in Canada?

- Yes
- No

***Consent form to Participate in a Research Study***

**Study Title: A Survey of Acceptability of Grab Bars in Homes**

**Investigator:**

Alison Novak, PhD (Principal Investigator)

Scientist

Toronto Rehabilitation Institute - University Health Network

**Contact Information:**

E-mail: **********

Telephone: ***********

Please note that the security of e-mail messages is not guaranteed. Messages may be forged, forwarded, kept indefinitely, or seen by others using the internet.

Do not use e-mail to discuss information you think is sensitive. Do not use e-mail in an emergency since e-mail may be delayed.

**Introduction:**

You are being asked to participate in this study because you are over the age of 18 and are a resident or citizen of Canada. This page describes the risks and benefits of participating. If you have any questions, please feel free to contact us. Participation in this study is voluntary.

**Background/Purpose:**

Slips, trips, and falls are important safety concerns for many people when getting in to and out of, and using their bathtub or shower stall. Grab bars have been shown to assist with preventing falls, although it is NOT mandatory to install a bathroom grab bar in your home.

We are conducting a research study on bathroom grab bars. You are being asked to participate because we want to learn about your views on installing and using grab bars in your bathroom. 700 individuals will be recruited to take part in the survey. In the survey, we will ask questions about you (e.g., age, gender, job sector) and about the use of assistive devices and bathroom grab bars. The survey takes approximately 3 to 5 minutes to complete.

**Risks:**

Taking part in this study has minimal risks. The questions in this survey have been designed so that the likelihood that the information can be linked to you and your identity is minimal. However, because this study is being delivered online, data security cannot be completely guaranteed.

**Benefits:**

This study collects information about public perceptions of grab bar installation and use that may be used to guide the development of building codes and standards and clinical recommendations. There is no direct, immediate benefit to you.

**Confidentiality:**

If you agree to take part in this survey, the Principal Investigator and her study team will collect only the information they need for the study. Data will be stored in a secure and confidential location for 10 years, after which it will be destroyed.

Representatives of the University Health Network (UHN) including the UHN Research Ethics Board may come to the hospital to look at the study records and personal information to check that the information collected for the study is correct and to make sure the study is following proper laws and guidelines. Your data will be shared as described in this consent form or as required by law. You will not be named in any reports, publications, or presentations that may come from this study. We are using SurveyMonkey.com to collect your responses to the survey. Your responses to the survey will be retained by SurveyMonkey until the research team closes the study. Additionally, SurveyMonkey may collect cookies in order to facilitate your survey experience. Please visit https://www.surveymonkey.com/mp/legal/privacy-policy/#pp-section-6 if you wish to understand what data SurveyMonkey collects, and how to modify your preferences regarding cookies.

**Voluntary Participation:**

Your participation in this survey is voluntary. You are free to choose to not complete the survey. You may decide to withdraw from the study at any time before the survey is submitted. After you have submitted the survey it is not possible to withdraw from the study.

**Costs and Reimbursement:**

There is no cost for you to participate in this survey. At the end of the survey, you will be have the opportunity to enter a draw for a $20 CAD gift card. One gift card will be issued for every 10 people that complete the survey.

**Rights as a Participant:**

By consenting to taking part in this study, you do not give up any of your legal rights against the investigators, sponsor or involved institutions for compensation, nor does this form relieve the investigators, sponsor or involved institutions of their legal and professional responsibilities.

**Conflict of Interest:**

Researchers have an interest in completing this study. Their interests should not influence your decision to participate in this study

**Questions about this Study:**

If you have any questions, concerns or would like to speak to the study team for any reason, please contact Alison Novak via e-mail at *********** or telephone at **********

If you have any questions about your rights as a research participant or have concerns about this study, call the Chair of the University Health Network Research Ethics Board (UHN REB) or the Research Ethics office number at 416-581-7849. The REB is a group of people who oversee the ethical conduct of research studies. The UHN REB is not part of the study team. Everything that you discuss will be kept confidential.

3. Do you consent to taking part in this study and agree to the use of your information * as described above?

- Yes
- No

***About You***

4. What is your age (in years)?

*Free answer*

5. What is your gender?

- Man
- Woman
- Other
- Prefer not to answer

6. Which job sector(s) do you primarily identify with? Please select all that apply.

- Agriculture, forestry, fishing and hunting, natural resource extraction
- Utilities
- Construction
- Manufacturing
- Wholesale and retail trade
- Transportation and warehousing
- Information and cultural industries, arts, and entertainment and recreation
- Finance and insurance, real estate and rental and leasing
- Professional, scientific and technical services
- Management of companies and enterprises
- Administrative and support, waste management and remediation services
- Education services
- Health care and social assistance
- Public administration
- Other services (except public administration)
- Other (please specify)

7. Do you have an impairment(s) in or difficulty with any of the following? Please select all that apply.

If you use any assistive devices or aids (e.g., glasses, hearing aids, mobility devices, etc.), please answer this question as if you were NOT using any assistive devices or aids.

- Mobility (e.g., difficulty with balance control, walking, and/or using stairs)
- Vision/Seeing
- Hearing
- Cognition/Memory (e.g., difficulty with remembering, concentrating, learning)
- Other (please specify)

8. What type of dwelling or home do you currently live in?

- A dwelling or home that I OWN
- A dwelling or home that I RENT
- A dwelling or home that I NEITHER OWN NOR RENT
- Other (please specify)

9. Where do you currently live?

- House (including detached, semi-detached, or row house)
- Apartment or condominium
- Mobile home or trailer
- Retirement home, long-term care home, or group home
- Other (please specify)

10. Do you have a grab bar in your bathroom?

- Yes
- No
- Prefer not to answer

11. Would you allow a grab bar to be installed if it was provided at no cost to you?

- Yes
- No
- Prefer not to answer

12. Would you OBJECT to grab bar installation becoming mandatory in newly built or newly renovated homes?

- Yes
- No
- Prefer not to answer

13. Would you use a grab bar if it was in your bathroom?

- Yes
- No
- Prefer not to answer
